# Impact of overproduced heterologous protein characteristics on physiological response in *Yarrowia lipolytica* steady-state-maintained continuous cultures

**DOI:** 10.1007/s00253-020-10937-w

**Published:** 2020-10-06

**Authors:** Paulina Korpys-Woźniak, Piotr Kubiak, Wojciech Białas, Ewelina Celińska

**Affiliations:** Department of Biotechnology and Food Microbiology, Poznan University of Life Sciences, ul. Wojska Polskiego 48, 60-627 Poznań, Poland

**Keywords:** Heterologous protein, Recombinant strain, Overexpression, Overproduction, Secretion, Chemostat, Steady state

## Abstract

Overproduction of recombinant secretory proteins triggers numerous physiological perturbations. Depending on a given heterologous protein characteristics, the producer cell is faced with different challenges which lead to varying responses in terms of its physiology and the target protein production rate. In the present study, we used steady-state-maintained *Yarrowia lipolytica* cells to investigate the impact of different heterologous proteins on the physiological behavior of the host cells. Such an approach allowed to uncouple the impact of the overproduction of a particular protein from the phenomena that result from growth phase or are caused by the heterogeneity of the analyzed populations. Altogether, eight variants of recombinant strains, individually overproducing heterologous proteins of varying molecular weight (27–65 kDa) and reporting activity (enzymatic and fluorescent) were subjected to chemostat cultivations. The steady-state-maintained cells were analyzed in terms of the substrate utilization, biomass and metabolites production, as well as the reporter protein synthesis. Simplified distribution of carbon and nitrogen between the respective products, as well as expression analysis of the heterologous genes were conducted. The here-obtained data suggest that using a more transcriptionally active promoter results in channeling more C flux towards the target protein, giving significantly higher specific amounts and production rates of the target polypeptide, at the cost of biomass accumulation, and with no significant impact on the polyols production. The extent of the reporter protein’s post-translational modifications, i.e., the number of disulfide bonds and glycosylation pattern, strongly impacts the synthesis process. Specific responses in terms of the protein formation kinetics, the gene expression levels, and transcript-to-protein linearity were observed.

**Key Points**

*• Eight expression systems, producing different reporter proteins were analyzed.*

*• The cells were maintained in steady-state by continuous chemostat culturing.*

*• Protein- and promoter-specific effects were observed.*

**Electronic supplementary material:**

The online version of this article (10.1007/s00253-020-10937-w) contains supplementary material, which is available to authorized users.

## Introduction

Growing demand for industrial enzymes and biopharmaceutical proteins triggers increasing interest in microbial cell factories that serve as protein production platforms. It is now well recognized that yeast-based expression systems offer a unique combination of the ease of manipulation with complex folding and maturation of the target polypeptide. Among several industrially relevant hosts, *Yarrowia lipolytica* emerges as a highly attractive system for heterologous protein production (Madzak 2018), which was enabled by the expansion of the synthetic biology toolbox designed for this species (Wagner and Alper 2016; Larroude et al. 2018; Park et al. 2019). Moreover, *Y. lipolytica* was found to perform well in bioreactor cultures in terms of cell growth, secretory protein titer, and productivity (Celińska et al. 2017a; Theron et al. 2020). It was evidenced that *Y. lipolytica* produces high levels of active enzyme, generates stable isotope-labelled variants of a secretory protein, or that it is suited for the production of complex high molecular weight proteins targeted for secretion (Madzak et al. 2005; Boonvitthya et al. 2013; Nars et al. 2014; Theron et al. 2020). Recently, *Y. lipolytica* was shown to manage secretory production of a heterologous protein of molecular weight > 120 [kDa] (Swietalski et al. 2020).

Overproduction of recombinant secretory proteins triggers numerous physiological perturbations to the host cell, frequently leading to decreased growth rate and underperformance in terms of the desired polypeptide production (Graf et al. 2009; Puxbaum et al. 2015). It is now well recognized that depending on the characteristics of a given heterologous protein, the producer cell is faced with different challenges, and that eventual bottlenecks and limitations may occur at different levels of the protein production/secretory pathway (Gasser et al. 2007; Nocon et al. 2014). Omics-assisted approaches allow to get deeper insight into actual processes taking place in the cell upon overproduction of a specific protein. It was demonstrated that over 60 genes were transcriptionally changed when either of two different heterologous proteins were overproduced in *Saccharomyces cerevisiae* (Liu et al. 2013). Correspondingly, different genetic engineering strategies allow to alleviate limitations encountered upon overproduction of different heterologous proteins (Hou et al. 2012; Puxbaum et al. 2015; Celińska and Nicaud 2019). It was highlighted that larger proteins, even if produced at moderate level, impose much higher metabolic burden to the producer cells than smaller ones (Liu et al. 2013). In that study, it was shown that upon overproduction of a larger secretory protein, the genes involved in the amino acid and energy metabolisms were significantly upregulated when compared with even more intensive production of a smaller protein. This leads to a conclusion that, depending on the overproduced protein characteristics, the response from the host cell in terms of its physiology and the production rate will be different.

The kinetics of production of a heterologous secretory protein and the host’s response to such a challenge can be most accurately studied in steady-state-maintained cells (Arvas et al. 2011; Liu et al. 2013; Peebo and Neubauer 2018). During a steady state, the concentration of intra- and extracellular molecules produced or metabolized by cultured cells remain constant over prolonged time which is counted in culture residence times (Hoskisson and Hobbs 2005). The steady state can be reached by synchronized feeding of nutrients and withdrawal of culture from a bioreactor vessel at a specified dilution rate (*D*; [h^−1^]). It was observed that a culture reaches the steady state by somehow matching its growth rate exactly to the dilution rate over a wide range of dilution rate values. One of the key advantages of such a mode of cultivation is that under the steady state, the population is reasonably homogenous and synchronized in a specific growth phase. Such approach eliminates a number of molecular phenomena related to growth phase, stress response, or the other types of heterogeneities occurring in a typical batch culture, which, if not controlled, may lead to incorrect interpretation of physiological changes occurring in the cell. Chemostat cultivations are relevant for both applied and basic research and are frequently used in studies on recombinant protein production in a variety of microbial expression platforms (e.g., *Escherichia coli*, *Komagataella phaffii*, *S. cerevisiae*, and *Aspergillus niger*) (Pedersen et al. 2000; Rodríguez-Carmona et al. 2012; Liu et al. 2013; Looser et al. 2014). So far, most of the research involving chemostats and *Y. lipolytica* was focused on the production of small molecular weight metabolites, such as citric acid, erythritol, and lipids (Rywińska et al. 2011; Ochoa-Estopier and Guillouet 2014; Rakicka et al. 2017), and no investigations into heterologous protein production kinetics under steady state have been conducted to date.

In the present study, we used continuous chemostat cultures to develop steady-state-maintained populations of *Y. lipolytica* recombinant strains producing four variants of reporter proteins—a fluorescent reporter in an intracellular and secreted form, and two enzymatic reporters targeted for secretion. The strains were constructed in two expression platforms, comprising the most popular *Y. lipolytica* expression hosts. Altogether, eight variants of recombinant strains, overproducing individually the heterologous proteins, and two reference strains were subjected to chemostat cultivations. Steady-state-maintained cells were analyzed in terms of the biomass and metabolite production, substrate utilization kinetics, and reporter protein production. Simplified distribution of carbon (C) and nitrogen (N) between the respective products and expression of the heterologous genes were analyzed as well.

## Materials and methods

### Strains and small-scale cultivations

All the strains and vectors used in this study are listed in Table S1. Regarding *Y. lipolytica* host strains used in this study, both Po1h and Po1g originated from the same wild-type strain (W29) and are deleted for both extracellular proteases (AEP and AXP). On the other hand, the strains differ in the auxotrophy selection marker (Leu2 for Po1g and Ura3 for Po1h), integration site of the recombinant cassette (pBR-platform and random integration via zeta elements), and the promoter-governing expression of the cloned genes (hp4d and 4UAS-pTEF, respectively). *Y. lipolytica* and *E. coli* strains were routinely maintained as described in Barth and Gaillardin (1996) and Sambrook and Russell (2001). Briefly, *Escherichia coli* strain JM109 was grown in LB medium ([g L^−1^]: yeast extract (BTL, Lodz, Poland), 5; bactopeptone (BTL), 10; NaCl (POCh, Gliwice, Poland), 5) supplemented with appropriate antibiotic (ampicillin at 100 [mg L^−1^] or kanamycin at 40 [μg L^−1^]) and agar ((Biomaxima, Lublin, Poland), 15 [g L^−1^]) at 37 °C and with 250 rpm shaking. *Y. lipolytica* strains were cultured in yeast nitrogen base (YNB; [g L^−1^]: YNB (Sigma-Aldrich, Merck KGaA, St. Louis, USA), 1.7; (NH_4_)_2_SO_4_ (PoCh), 5; glucose (PoCh), 20), yeast extract-peptone-dextrose (YPD; [g L^−1^]: yeast extract, 10; bacto peptone, 20; glucose, 20), or yeast extract-peptone-starch (YPS; [g L^−1^]: yeast extract, 10; bacto peptone, 20; starch (Sigma-Aldrich)), 10; glucose, 20) solidified with agar (15 [g L^−1^]), at 30 °C and with 250 rpm shaking.

### Molecular biology protocols

Standard molecular biology techniques were used throughout the research (Barth and Gaillardin 1996; Sambrook and Russell 2001). Manipulations with DNA fragments were conducted using appropriate kits from A&A Biotechnology (Gdynia, Poland). DNA was amplified using Phire DNA polymerase (Thermo Fisher Scientific, Waltham, MA USA) or RUN DNA polymerase (A&A Biotechnology). Two *Y. lipolytica* strains were used as hosts in this study: Po1g and Po1h (Table S1). Three genes encoding recombinant proteins were expressed individually in each host (Table S2), as follows: (i) enzymatic reporter: α-amylase (SoA) derived from rice weevil *Sitophilus oryzae* (52 [kDa]), (ii) enzymatic reporter glucoamylase (TlG) from fungus *Thermomyces lanuginosus* (65 [kDa]), and (iii) fluorescent reporter: YFP (Yellow Fluorescent Protein, YFP) (27 [kDa]). The last reporter was cloned as either intracellular (inYFP) or secreted protein (scYFP). Altogether, four variants of the reporters were used: scSoA, scTlG, scYFP, inYFP. According to our previous studies, neither SoA nor TlG activity could be detected inside the *Y. lipolytica* host cells (unpublished), making inSoA/inTlG variants pointless.

**Fig. 1:**
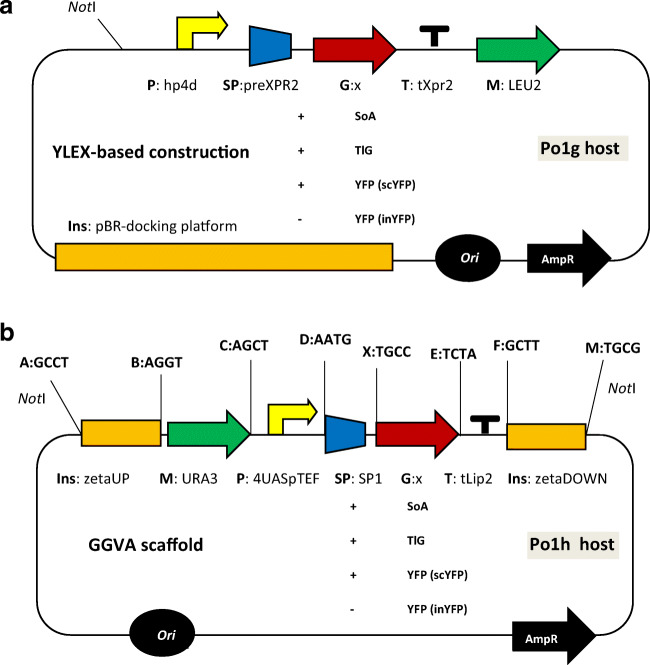
Cloning vectors used in this study. **a** YLEX commercial cloning system-based vectors. **b** Golden Gate assembly-based vectors. The GGVA was assembled using a set of 4-nt overhangs indicated in the scheme (A, B, C, D, X, E, F, and M). Abbreviations: Ins, regions for non-homologous recombination (GGVA) or directing integration with pBR-docking platform (YLEX); M, selection marker LEU2/URA3; P, promoter 4UAS-pTEF/hp4d; SP, signal peptide (derived from spYALI0B03564g for GGA or XPR2 gene for YLEX-based expression); G, target gene (one of the three SoA/TlG/YFP devoid of their native SP); T, terminator tLip2/tXpr2; ori, bacterial origin of replication; AmpR, bacterial selection marker. The expression cassettes were digested with *Not*I restriction endonuclease prior to transformation

In *Y. lipolytica* Po1g host, the genes were cloned using a commercial cloning system (YLEX™ kit; Yeastern Biotech Co. Ltd; Fig. 1a), integrating the expression cassettes in a pBR322 docking platform. For the secretion, the genes were transcriptionally fused with preXPR2 signal sequence. Expression of the heterologous genes was governed by a strong, semi-constitutive promoter hp4d (Madzak et al. 2000). In *Y. lipolytica* Po1h host strain, the genes were cloned using Golden Gate expression cassettes bearing a single transcription unit (TU) (Celińska et al. 2017b) (Fig. 1b), under the control of a strong, semi-constitutive 4UAS-pTEF promoter (Dulermo et al. 2017). The reactions were conducted using T4 ligase and *Bsa*I restriction endonuclease from New England Biolabs (NEB, Ipswich, MA, USA). For the secretion, the genes were transcriptionally fused with a SP1 signal sequence, native for exo-1,3-beta-glucanase (YALI0B03564g) (Celińska et al. 2018). The expression cassette assembly was conducted as described previously (Celińska et al. 2017b). All the clonings were conducted using primers listed in Table S3.

The expression cassettes were sequenced (Genomed, Warsaw, Poland) to verify their correctness, linearized with *Not*I (Thermo Fisher Scientific) and transformed into Po1h and Po1g *Y. lipolytica* strains. Clones appearing after 48 h were replicated on a fresh YNB plate and verified for the presence of the introduced heterologous genes through colony PCR with specific primers (Table S3). Acquired amylolytic phenotypes (scSoA, scTlG) were screened via starch-iodine drop test on YPS agar plates, as described previously (Celińska et al. 2016). Acquired fluorescence phenotypes (inYFP, scYFP) was screened using AxioVert fluorescence microscope (ZEISS, Oberkochen, Germany) and quantitatively assessed in microcultures using an automatic plate reader/fluorimeter (Tecan Infinite M200; Tecan Group Ltd., Männedorf, Switzerland). Positive strains were deposited as glycerol stocks at − 80 °C.

### Shake-flask cultures for selection of representative sub-clones

Three sub-clones were randomly selected from amongst the positive *Y. lipolytica* transformants. The sub-clones were subjected to primary screening to select representative strains for further studies. Screening cultures were conducted in 50 mL shake flasks containing 5 mL of YPG_20_ medium ([g L^−1^]: yeast extract, 10; peptone, 20, glycerol, 20) at 28 °C and 250 rpm shaking for 48 h. Samples were periodically withdrawn from the cultures and, after centrifugation, the biomass and the supernatant samples were stored separately, at − 20 °C, until analyzed. After thawing, the biomass was washed twice in sterile saline solution (0.85% NaCl) and appropriately diluted cell suspensions were subjected to spectrophotometric measurements at 600 nm wavelength (Tecan Infinite M200).

### Inoculum preparation

Selected, representative sub-clones were revived by transferring the biomass from the glycerol stock onto YPD agar plate and incubating at 30 °C for 24 h. Single colonies were transferred into 100 mL shake flasks containing 30 mL of YPG_20_ medium and cultured for 22 h, at 28 °C and 250 rpm in an orbital shaker incubator (BIOSAN, ES-20, Riga, Latvia). Such pre-cultures (30 mL) were aseptically transferred into a bioreactor vessel (10% of the bioreactor working volume).

### Bioreactor cultivations

Batch and continuous cultures were performed in a 0.5-L stirred tank Multifors 2 bioreactor (Infors HT, Bottmingen-Basel, Switzerland) equipped with two flat-bladed turbines with six blades (Rushton turbine). Throughout the cultures, the temperature, pH and dissolved oxygen (DO; [%]) were stabilized at 28 °C, pH 5.5 by automatic addition of 20% NaOH, 10% H_2_SO_4_, and at 20 [%] (oxygen saturation) by using cascade-control approach with stirring from 100 up to 1200 rpm, and constant aeration at 2.0 vvm. Foaming was minimized by the addition of a defoaming agent AntiFoam 204 (Sigma-Aldrich). The cultures were conducted at a working volume of 0.3 L and were initiated with a batch culture stage in YPG_100_ medium ([g L^−1^]: yeast extract, 10; bactopeptone, 20; glycerol, 100). Continuous cultivation stage was initiated when glycerol was nearly completely consumed, which was indicated by an increase in the DO parameter. Continuous stage was executed by constantly feeding YPG_100_ medium and withdrawing an equal volume of the culture at a working dilution rate [*D*; h^−1^] of approximately 0.20 [h^−1^]. The *D* value was determined in preliminary studies. The steady state, marked by constant concentration of the biomass, residual glycerol, and metabolites, was reached after at least six residence times [nV]. Two milliliter samples were collected periodically, centrifuged at 12,045×*g* for 6 min (Eppendorf MiniSpin; Hamburg, Germany) and stored at − 20 °C until analyzed, except for the YFP-containing samples. For the YFP-producing strains, the biomass was washed in sterile saline solution and resuspended in the equal volume of the same solution prior to storage. The supernatants and the washed biomass were stored at -80 °C for further analyses. All the cultures were conducted in at least two biological replicates.

### Analytical methods

#### Biomass concentration and cell viability

The biomass samples were defrosted and washed twice with a sterile saline solution. Dry cellular weight (DCW; [g_DCW_ L^−1^]) was determined gravimetrically through drying the biomass in a laboratory dryer at 105 °C for 2 - 3 days, until a stable readout was reached. The dry biomass concentration was expressed in grams of the cell dry mass per liter [g_DCW_ L^−1^]. The strains viability during the bioreactor cultivations was assessed by surface-plating and colony counts. Upon sampling, the cultures were decimally diluted in a sterile saline solution in MTP up to 10^10^-fold, followed by plating on YPD agar plates using a stainless steel replicator (Sigma-Aldrich). The plates were incubated for 24 h at 30 °C. The results were expressed as surface colony forming units [scfu] reflecting the number of colony counts at the highest decimal dilution, where the colony growth was observed.

#### Concentration of the substrate and metabolites determined by HPLC

The culture supernatants were thawed on ice, diluted, and passed through 0.45 μm membrane syringe filters (Millipore; Merck-Millipore, Burlington, MA, USA). A high-performance liquid chromatograph, Agilent Technologies 1200 series (Agilent Technologies, Santa Clara, CA, USA), was used to determine the concentration of glycerol (GLY; [g L^−1^]) and metabolites (erythritol, ERY; mannitol, MAN; citric acid, CA; α-ketoglutaric acid, α-KG [g L^−1^]) contained in the culture liquid. The apparatus was equipped with a refractive-index detector (G1362A) and a Rezex ROA-Organic Acid H+ column (Phenomenex, Torrance, CA, USA). Operating conditions were as follows: 0.005 N H_2_SO_4_ as eluent at a flow rate of 0.6 [mL min^−1^]; the column temperature was set at 40 °C. External standards (purchased from Sigma-Aldrich) were used for identification and quantification of the peaks areas in chromatograms, which were analyzed using ChemStation for LC 3D software (Agilent).

#### Fluorescence analysis

All the fluorescence measurements were conducted in flat-bottomed MTP plates (Corning; Sigma-Aldrich) in Tecan Infinite M200 automatic plate reader at the following wavelength settings: excitation at 495 nm/emission at 527 nm. The extracellular YFP fluorescence was measured in 200 μL of the supernatant samples defrosted on ice. Fresh culture medium (also stored at − 80 °C) was used as the background reference. The intracellular YFP fluorescence was measured using the pre-washed biomass samples, thawed on ice and resuspended in sterile saline solution. Serial dilutions were conducted prior to the measurements. The reference strains (Po1g_Leu2+ and Po1h_Ura3+) were used for the background normalization. Importantly, the pre-washed biomass of the reference strains did not exhibit intrinsic, background fluorescence under the adopted assaying conditions. All the measurements were conducted in technical triplicate out of each biological duplicate. After normalization, the fluorescence results were expressed in relative fluorescence units [RFU]. RFU was defined as the sample median fluorescence value minus the background fluorescence value (fresh medium or the reference strains biomass). Additionally, the fluorimetry results were randomly verified through observations under fluorescence microscope (ZEISS AxioVert, AxioCam 350 color; filterset: 09; ZEISS).

#### Enzymatic assays—SoA-microSIT and TlG-microDNS

Miniaturized colorimetric assays were used to determine the extracellular activity of both enzymatic reporters. The SoA amylolytic activity was examined in the supernatant samples using microSIT assay, described previously (Borkowska et al. 2019). One activity unit (α-amylase activity unit [AAU]) refers to the amount of enzyme that decomposes 1 mg of starch per 1 mL, during 1 min, at pH 5.0 and 40 °C, under the applied experimental conditions. The TlG glucoamylase activity was examined in the supernatant samples using microDNS (3,5-dinitrosalicylic acid) method according to (Goncalves et al. 2010). One activity unit (glucoamylase activity unit [GAU]) was defined as the amount of enzyme that releases 1 μg of reducing sugar equivalents per 1 mL, during 1 h, at pH 5.0 and 40 °C, under the adopted assay conditions. In both enzymatic assays, the reference strains Po1h_Ura3+/Po1g_Leu2+ were assayed under the same conditions to assess any possible background activity. All the readouts were normalized per blank reaction with distilled water. All the colorimetric enzymatic assays were read using the Tecan Infinite M200 plate reader at 580 nm (microSIT) and 540 nm (microDNS) wavelength. Each sample was analyzed in technical duplicates.

#### Heterologous gene expression analysis through RTqPCR

Expression level of the target genes (SoA, TlG, YFP) was analyzed in the samples withdrawn from the cultures in the steady state. The biomass from 2 mL of the culture was used for isolation of total RNA. RNA isolation was performed using Bead-Beat Total RNA Mini Kit (A&A Biotechnology). Isolated RNA was verified for quantity and quality through gel electrophoresis and spectrophotometric measurement (NanoDrop, Thermo Fisher Scientific). cDNA synthesis was conducted using SuperScript III Reverse Transcriptase and oligo(dT) primer, according to the manufacturer’s protocol (Thermo Fisher Scientific). cDNA preparations were used as templates in RTqPCR, carried out in an Applied Biosystems 7500 device (Applied Biosystems, Foster City, USA). The reactions were set up using RT HSPCR Mix SYBR® B (A&A Biotechnology) according to the manufacturer’s specifications. LoROX dye was used as a passive reference. The expression level of the genes was normalized to the expression level of the actin gene (*ACT1*), used as internal calibrator. All the primers for RTqPCR were designed with Primer Expert Software (Applied Biosystems) and are listed in Table S3. The following thermal profile was used: 95 °C 3 min, (95 °C 15 s, 60 °C 30 s, 72 °C 30 s) × 40, 72 °C 1 min, Melt Curve 94 °C 15 s, 60 °C 60 s, 95 °C 30 s, 60 °C 15 s. Fluorescence from SYBR®Green was measured at the end of the elongation step, and obtained data were processed according to ΔΔCT method (Livak and Schmittgen 2001). cDNA preparations of the reference strains (*Y. lipolytica* Po1h_Ura3+ and Po1g_Leu2+) were used as the external calibrators, to which 1.0 expression level was assigned. All the samples were analyzed in technical duplicates.

### Data processing and statistical analysis

#### Concentration of the reporter proteins

The absolute concentration of enzymatic reporters was calculated based on their specific activity values, equal to 80 [GAU mg^−1^] for TlG (Basaveswara Rao et al. 1981) and 478 [AAU mg^−1^] for SoA (Baker and Woo 1985). The absolute concentration of eYFP reporter was calculated based on a calibration curve for eGFP provided by (BioTek Instruments, Inc., Winooski, VT, USA). For the purpose of re-calculation, the following data were used to estimate the relative brightness of eYFP with respect to eGFP: *ε*_eYFP_ = 83,400 [cm^−1^M^−1^], *Φ*_f eYFP_ = 0.61, *ε*_eGFP_ = 55,000 [cm^−1^M^−1^], *Φ*_f eGFP_ = 0.60 (Wall et al. 2015).

#### Simplified carbon and nitrogen distribution between the products

Distribution of C and N was calculated based on molecular weight and elementary composition of the major products synthesized by *Y. lipolytica* strains in the chemostat cultures. The following formulas and molecular weight values were used to calculate the content of carbon and nitrogen in the target protein: TlG: C_2910_H_4365_N_781_O_909_S_10_, and 65,154.96 [Da]; SoA: C_2299_H_3433_N_629_O_730_S_18_, and 52,140.40 [Da]; eYFP: C_1215_H_1863_N_317_O_364_S_8_, and 26,991.54 [Da]. All these data were withdrawn from ExPASy server, using ProtParam tool (Gasteiger et al. 2005). Elementary composition of *Y. lipolytica* biomass was defined previously (Celińska et al. 2017a). A sum of C and N contained in all the products considered in this analysis was defined as 100%, and a fraction of each particular product was calculated based on determined concentration of DCW and the metabolites and the amount of the target protein, calculated as indicated above.

#### Statistical analysis

Statistical analysis was performed using Statistica 13 software (Tibco, CA, USA). After confirming homogeneity of variance, Tukey’s HSD test was used to identify statistically homogenous groups of data at *p* < 0.05. Error bars indicate ±SD of at least three replicates.

## Results

### Construction and selection of representative clones

Several positive sub-clones received after transformation of the parental strains were grown in shake-flasks to investigate inter-clonal variation and to identify possible growth impairment due to the genetic modification. As shown in Fig. S1, in the majority of cases, the observed variation in growth was negligible between the sub-clones and the reference strains (Po1h_Ura3+ or Po1g_Leu2+). In all the cases, it was possible to select a sub-clone being representative for each of the variants, demonstrating identical growth as the corresponding reference strain. These strains were subjected to the following studies.

### Dilution rate adjustment

Prior to setting the bioreactor chemostat cultivations with the selected representative sub-clones, preliminary cultivations of the reference strain Po1h_Ura3+ were conducted to determine appropriate dilution rate of the culture. Three dilution rate settings were tested: 0.06, 0.12, 0.20 [h^−1^]. Two parameters were used as determinants of the steady state: concentration of DCW [g_DCW_ L^−1^] and concentration of GLY [g L^−1^]. The results are shown in Fig. S2. At the lowest *D*, none of the determinants could be stabilized, demonstrating fluctuations from 56.37 to 75.53 and 14.40 to 23.85 [g L^−1^] in GLY and DCW concentrations, respectively. Such high variation in DCW and GLY indicate high heterogeneity of the cultured cells population in terms of growth phases. Increasing D to 0.12 [h^−1^] resulted in similar lack of stabilization of DCW, which fluctuated from 16.90 to 26.65 [g L^−1^]. Accordingly, GLY concentration varied between 78.32 and 94.34 [g L^−1^], which was still not satisfactory. The highest D tested, equal to 0.20 [h^−1^], allowed to reach sufficiently stable readouts in both steady-state determinants, 88.71 to 91.05 of GLY [g L^−1^] and 8.15 to 10.35 DCW [g_DCW_ L^−1^]. Additionally, all the remaining metabolites were present in the culturing media at stable concentrations. Moreover, based on the results of this experiment it can be stated that oxygen availability was the growth-limiting factor. At D 0.20 [h^−1^], the DO [%] was maintained near 20 [%] without extreme changes in the stirring rate. At dilution rates of 0.06 and 0.12 [h^−1^], the readouts from the DO probe indicated complete oxygen depletion, making it impossible to precisely control DO in the bioreactor which might have triggered the lack of long-term stability of the cultures. The outcome indicates that at the D value of 0.2 [h^−1^] the cultured population was homogenous in terms of growth phase and that the steady state was reached. Therefore, this value of D was used in the following chemostat cultivations of the selected representative *Y. lipolytica* sub-clones and the reference strains.

### Chemostat cultivations—general observations

**Fig. 2:**
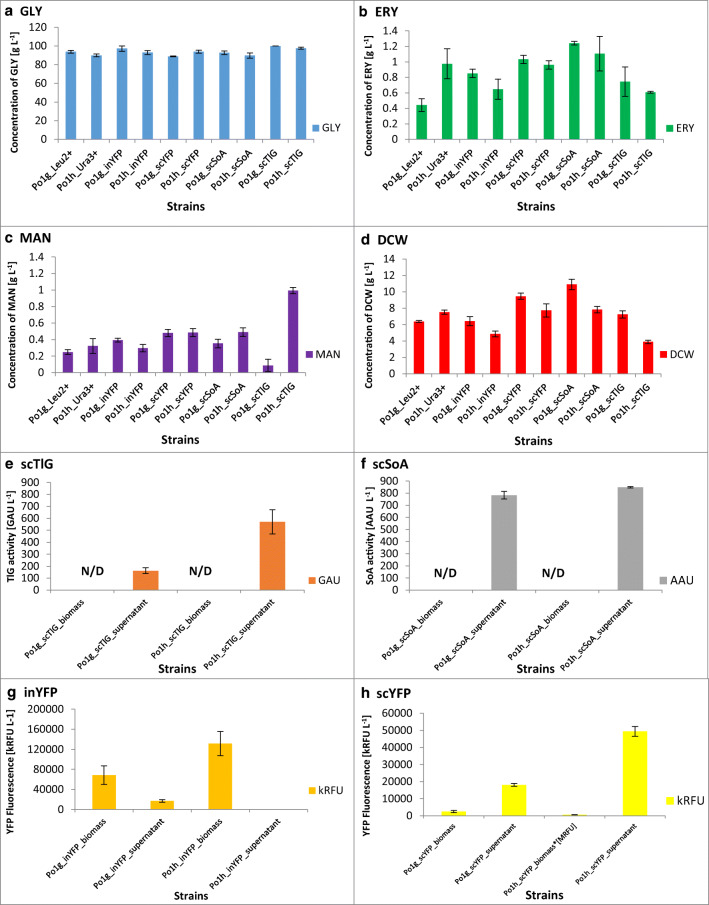
Glycerol consumption, concentration of major small molecular weight metabolites, biomass, secretory protein production (scTlG/scSoA), and yellow fluorescence (inYFP/scYFP) during steady-state chemostat cultivations of Po1g- and Po1h-derived *Yarrowia lipolytica* strains. *x*-axis: strains. *y*-axis: **a** concentration of glycerol (GLY) in [g L^−1^], **b** concentration of erythritol (ERY) in [g L^−1^], **c** concentration of mannitol (MAN) in [g L^−1^], **d** biomass accumulation: dry cell weight (DCW) in [g_DCW_ L^−1^], **e** TlG activity in culture medium in [GAU L^−1^], **f** SoA activity in culture medium in [AAU L^−1^], **g** YFP fluorescence of Po1g_inYFP/Po1h_inYFP recombinant strains in the cellular pellet and culture medium in [kRFU], **h** YFP fluorescence of Po1g_scYFP/Po1h_scYFP recombinant strains in the cellular pellet and culture medium in [kRFU]. Error bars indicate ±SD from at least biological duplicates in two technical replicates. N/D, non-detected

All the chemostat cultivations were conducted according to the same scheme—feeding of the medium and withdrawal of the culture were initiated immediately at the end of the batch stage (exhaustion of GLY marked by an increase in the DO [%] parameter). The steady state was reached typically after 6 to 9 residence times [nV]. The samples withdrawn from the cultures were analyzed for the concentration of the main carbon source, DCW and the key metabolites, viability of the cells, as well as the activity or fluorescence [AAU/GAU/RFU] resulting from expression of the respective heterologous genes. In Fig. 2, Table 1, and Table S4, these values were presented for all the strains maintained in the steady state, while Fig. S3 depicts kinetics of the whole cultures, including both the initial batch stage and the following chemostat stage. In the steady-state stage, GLY concentration was maintained at approx. 90 [g L^−1^] ensuring satisfactory provision of the carbon source for intensive production of the heterologous proteins (Fig. 2a; Fig. S3). While generally, the differences in GLY [g L^−1^] concentration were minor between the cultures of different strains, statistically important (*p* < 0.05) higher values were observed for Po1g- and Po1h-derived strains producing scTlG and Po1g-descendant producing inYFP, which was accompanied by a 2.82-, 2.71, and 1.96-fold lower glycerol consumption rate (*Q*_GLY_ ) than in the corresponding control strains (Table 1). Po1g derivatives producing scTlG, inYFP, and, additionally, scSoA were also characterized by a 3.09-fold, 2.00-, and 1.70-fold lower specific GLY utilization rate than in control strain (*p* < 0.05). As observed, the Po1g derivatives, producing heterologous proteins, were in majority characterized by lower specific GLY consumption (*Y*_s/x_ < 1.53 [g g_DCW_^−1^]) and specific GLY consumption rate (*q*_GLY_ < 0.20 ± 0.03 [g g_DCW_^−1^ h^−1^]) than their Po1h counterparts (except Po1h_scYFP). Alongside, the steady-state biomass concentration in those Po1g derivatives was higher in than in their Po1h-derived counterparts (Fig. 2d; Table 1; *p* < 0.05). Indeed, the highest biomass concentration and production rate was observed for Po1g-descendants producing scSoA and scYFP (*p* < 0.05). Significantly lower biomass concentration [g_DCW_ L^−1^] and production rate [g_DCW_ L^−1^ h^−1^] were observed for Po1h_scTlG (1.93- and 2.18-fold than the reference) and Po1h_inYFP (1.55- and 1.97-fold than the reference) (*p* < 0.05). Considering the absolute concentrations of the polyols produced by *Y. lipolytica* strains, their levels remained low (< 1.30 [g L^−1^]; Fig. 2b, c; Table 1), but some of the strains differed significantly from the remaining in terms of the polyols production, e.g., the highest ERY [g L^−1^] production was observed for Po1g_scSoA, while the lowest—for Po1h_scTlG, Po1h_inYFP, and Po1g_Leu2+ reference strain. As observed, the ERY concentrations were correlated with DCW values, as illustrated by highly corresponding specific ERY production rate (the highest for Po1h_scTlG 0.033 ± 0.002 [g g_DCW_^−1^ h^−1^], and the lowest for Po1g_Leu2+ 0.015 ± 0.003 [g g_DCW_^−1^ h^−1^]; all the remaining strains were grouped in the same homogenous group in HSD Tukey test at *p* < 0.05. MAN synthesis in the steady state was significantly different for two recombinant strains expressing TlG enzymatic reporter, for which the highest (Po1h_scTlG 0.99 ± 0.04 [g L^−1^], 0.054 ± 0.001 [g g_DCW_^−1^ h^−1^]) and the lowest (Po1g_scTlG 0.09 ± 0.08 [g L^−1^], 0.003 ± 0.002 [g g_DCW_^−1^ h^−1^]) MAN concentrations and specific production rates were observed. In this regard, Po1g_scTlG showed significantly different ratio of ERY to MAN formation reaching 12-fold higher production of the former (*p* < 0.05).

**Table 1:** The main bioprocessing parameters of the chemostat cultivations of *Yarrowia lipolytica*-derivatives producing the reporter proteins (inYFP/scYFP/scSoA/scTlG)

Strains	*Q*_GLY_ [g L^−1^ h^−1^]		*q*_GLY_ [g g_DCW_^−1^h^−1^]		*Q*_DCW_ [g L^−1^h^−1^]		*Y*_s/x_ [g g_DCW_^−1^]		*Y*_Protein/x_ [(kRFU/AAU/GAU) g_DCW_^−1^]		*Q*_protein_ [(kRFU/AAU/GAU) L^−1^ h^−1^]		*q*_protein_ [(kRFU/AAU/GAU) g_DCW_^−1^ h^−1^]	
	Mean	±SD	Mean	±SD	Mean	±SD	Mean	±SD	Mean	±SD	Mean	±SD	Mean	±SD
Po1g_Leu2+	2.14	0.35	0.34	0.05	1.43	0.03	1.50	0.23						
Po1h_Ura3+	3.28	0.07	0.44	0.01	1.81	0.03	1.86	0.11						
Po1g_inYFP	1.09*	0.52*	0.17*	0.07*	1.14*	0.09*	0.94	0.38	10,561.29 [kRFU g_DCW_^−1^]	1997.53	13,708.69 [kRFU L^−1^ h^−1^]	3359.42	2119.68 [kRFU g_DCW_^−1^ h^−1^]	370.76
Po1h_inYFP	1.95*	0.41*	0.40	0.08	0.92*	0.07*	2.13	0.43	27,680.92 [kRFU g_DCW_^−1^]	2893.94	24,343.02 [kRFU L^−1^ h^−1^]	4520.57	5163.48 [kRFU g_DCW_^−1^ h^−1^]	541.18
Po1g_scYFP	2.88	0.08	0.30	0.02	1.88*	0.07*	1.53	0.08	1390.96 [kRFU g_DCW_^−1^]	102.33	5286.58 [kRFU L^−1^ h^−1^]	220.50	540.81 [kRFU g_DCW_^−1^ h^−1^]	35.49
Po1h_scYFP	1.86*	0.32*	0.24*	0.03*	1.52*	0.16*	1.27	0.06	6387.61 [kRFU g_DCW_^−1^]	377.80	9440.37 [kRFU L^−1^ h^−1^]	540.61	1220.64 [kRFU g_DCW_^−1^ h^−1^]	58.86
Po1g_scSoA	2.18	0.39	0.20	0.03	2.24*	0.13*	0.97	0.16	71.58 [AAU g_DCW_^−1^]	4.15	162.07 [AAU L^−1^ h^−1^]	6.06	14.9 [AAU g_DCW_^−1^ h^−1^]	1.24
Po1h_scSoA	2.49	0.53	0.32	0.05	1.44*	0.07*	1.73	0.04	108.64 [AAU g_DCW_^−1^]	3.63	155.64 [AAU L^−1^ h^−1^]	0.91	19.92 [AAU g_DCW_^−1^ h^−1^]	0.54
Po1g_scTlG	0.76*	0.00*	0.11*	0.01*	1.65	0.10	0.47*	0.03*	22.73 [GAU g_DCW_^−1^]	5.65	57.99 [GAU L^−1^ h^−1^]	5.94	7.96 [GAU g_DCW_^−1^ h^−1^]	1.06
Po1h_scTlG	1.21*	0.22*	0.31	0.06	0.83*	0.04*	1.47	0.29	152.02 [GAU g_DCW_^−1^]	17.32	119.78 [GAU L^−1^ h^−1^]	18.24	32.08 [GAU g_DCW_^−1^ h^−1^]	4.07

*±SD*, standard deviation from at least two biological replicates and a specified number of technical replicates

*p < 0.05, significantly different from the references

Production of the reporter proteins expressed as total AU or FU per culture volume is presented in Fig. 2e–h and Table 1. No enzymatic activity AAU or GAU was detected in the yeast biomass (done for randomly selected samples), but only in the supernatants of scTlG (3.52-fold higher [GAU L^−1^] for Po1h than for Po1g derivatives) and scSoA (comparable [AAU L^−1^] for Po1h than for Po1g derivatives) producing strains. Some minor fluorescence level was observed in the culture medium of the Po1g strain producing inYFP, which was not the case for Po1h-derivative-producing inYFP without the signal peptide. On the other hand, a very high level of intracellular fluorescence was observed for Po1h_scYFP strain, producing YFP equipped with an operable signal peptide, which was 5-fold higher than for Po1h_inYFP, and 10-fold higher than for Po1g_inYFP. Nevertheless, the extracellular fluorescence for this strain was also the highest from those observed in this study; giving more than 2.5-fold higher RFU than the one by po1g_scYFP. For the latter strain, also the intracellular fluorescence was relatively low. In terms of specific activity or specific fluorescence measures [(kRFU/AAU/GAU) g_DCW_^−1^], Po1h derivatives exhibited superiority over Po1g derivatives, ranging from 1.51-fold [AAU g_DCW_^−1^], through 2.62- and 4.59-fold [RFU g_DCW_^−1^] for inYFP and scYFP, up to 6.69-fold [GAU g_DCW_^−1^] higher specific measure for the former (Table 1). Similar trends were observed for the volumetric productivity [(kRFU/AAU/GAU) L^−1^ h^−1^] and the specific productivity [(kRFU/AAU/GAU) g_DCW_^−1^ h^−1^] parameters (Table 1). The only example escaping this conclusion is the volumetric productivity of SoA which was comparable for Po1g- and Po1h-derived strains.

### Production of the reporter proteins in the chemostat cultures—comparison of different reporters

**Fig. 3:**
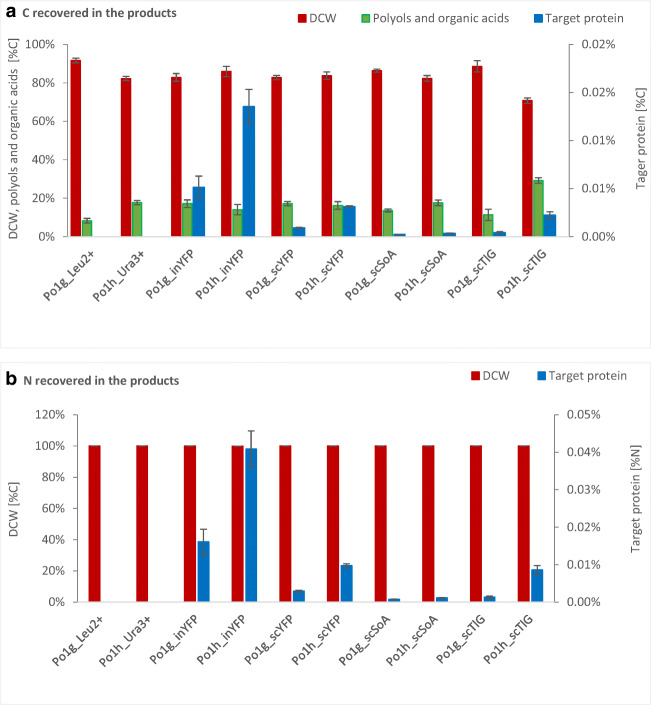
Simplified carbon and nitrogen distribution between the products. C and N amounts were determined based on either elementary analysis or chemical formulas. Total of C and N in all the considered products was defined as 100%. Error bars indicate ±SD from at least biological duplicates in two technical replicates

In order to directly compare production of different reporter proteins studied here, it was necessary to unify expression of their amounts to a uniform unit. Therefore, based on available specific activities of SoA and TlG, as well as calibration curve, molar extinction coefficient, brightness, and quantum yield of eGFP and eYFP, the amounts of the reporter proteins were estimated and expressed in grams and moles (Table 2). Noteworthy, it needs to be stressed that the following measures are just an approximation, and accurate comparisons can be done only for the variants with the same reporter protein (inYFP and scYFP together, separately SoA, and separately TlG). Irrespective of the adopted measure ([μg L^−1^], [pmol], [μg g_DCW_^−1^], [pmol g^−1^ h^−1^], etc.), Po1h_inYFP was superior in terms of the amount of produced reporter protein (*p* < 0.05), immediately followed by Po1g_inYFP. It was also reflected by the % of carbon (C) and nitrogen (N) channeled to the specific products synthesized by the *Y. lipolytica* strains under steady state (Fig. 3). In terms of the protein amounts expressed in molar values—[pmol] or [pmol h^−1^] or in the amount of C/N channeled to these products, it was observed that Po1h_scYFP produced significantly higher amounts of the reporter than any other strain producing secretory reporters (*p* < 0.05). Interestingly, in terms of the absolute molar amount [pmol] and volumetric productivity in moles [pmol h^−1^], a clear separation between the efficiency in production of the small reporter proteins (inYFP, scYFP) from the larger secretory reporters (SoA, TlG) was observed (*p* < 0.05). In the majority of measures, production of SoA by either of the host strains was the lowest; and usually, these two strains, Po1h_scSoA and Po1g_scSoA, were classified as the least efficient. Nevertheless, since all of the strains giving the highest/lowest absolute amounts of the reporter proteins synthesize YFP/SoA, it cannot be excluded that this observation results from the mode of the proteins amounts quantitation (calibration curve for YFP and specific activity for SoA and TlG). Only purification of the reporter proteins, experimental determination of their activity/fluorescence and amount in all the samples could provide accurate data in this regard, which is beyond the scope of this work. In every parameter and for all the reporter proteins studied here, Po1h turned out to be a superior host for the protein reporter production compared with Po1g (*p* < 0.05). This difference was the least clear for SoA.

**Table 2:** Estimated amounts and related production parameters of the reporter proteins (inYFP/scYFP/scSoA/scTlG) in continuous cultures of *Yarrowia lipolytica* derivatives

Strains	Protein [μg L^−1^]		Protein [pmol L^−1^]		*Q*_Protein [μg L^−1^ h^−1^]		*Q*_Protein [pmol L^−1^ h^−1^]		*Y*_Prot/X_ [μg g_DCW_^−1^]		*Y*_Prot/X_ [pmol g^−1^]		*q*_Prot_ [μg g^−1^ h^−1^]		*q*_Prot_ [pmol g^−1^ h^−1^]	
	Mean	±SD	Mean	±SD	Mean	±SD	Mean	±SD	Mean	±SD	Mean	±SD	Mean	±SD	Mean	±SD
Po1g_inYFP	375.93	105.14	13,927.33	3895.70	66.54	18.61	2465.00	689.59	58.06	12.19	2150.95	451.69	10.28	2.16	380.72	79.95
Po1h_inYFP	722.43	135.59	26,766.00	5024.37	136.54	25.63	5058.67	949.87	147.69	17.52	5471.85	649.13	27.91	3.31	1034.18	122.69
Po1g_scYFP	99.43	6.68	3684.67	248.47	19.79	1.33	733.33	49.33	10.53	0.94	390.00	34.74	2.10	0.18	77.61	6.92
Po1h_scYFP	271.30	18.37	10,051.67	680.18	53.17	3.60	1970.00	133.59	35.18	1.71	1303.27	63.39	6.90	0.33	255.44	12.43
Po1g_scSoA	28.87	1.19	553.00	22.72	5.92	0.24	113.33	4.73	2.65	0.23	50.90	4.50	0.54	0.05	10.44	0.92
Po1h_scSoA	31.23	0.15	599.33	3.51	5.75	0.03	110.33	0.58	4.00	0.21	76.68	4.02	0.74	0.04	14.11	0.74
Po1g_scTlG	33.77	5.15	518.00	78.94	7.67	1.17	118.00	17.69	4.69	1.01	72.02	15.48	1.06	0.23	16.35	3.52
Po1h_scTlG	118.83	21.03	1824.00	323.01	25.19	4.46	386.67	68.12	30.37	4.10	466.16	62.98	6.44	0.87	98.83	13.35

*±SD*, standard deviation from at least two biological replicates and a specified number of technical replicates

### Gene expression

**Fig. 4:**
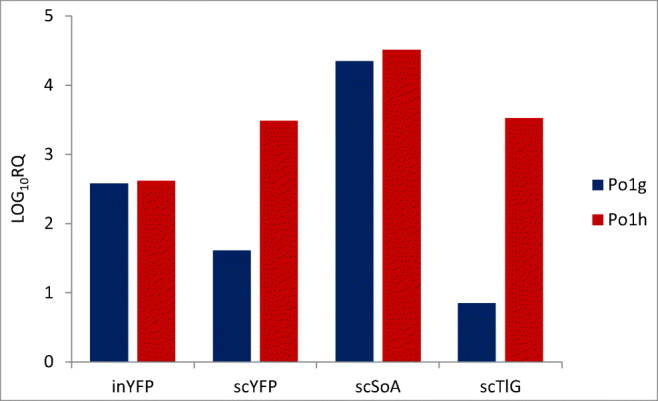
Relative expression levels of the heterologous genes encoding the reporter proteins in steady-state chemostat cultivations of *Y. lipolytica* recombinant strains. *x*-axis, reporter genes (scSoA, scTlG, inYFP, and scYFP); *y*-axis, LOG_10_RQ - Relative Quantitation values obtained in RTqPCR transformed logarithmically for more convenient presentation. Red: Po1h-derived *Y. lipolytica* strains. Blue: Po1g-derived *Y. lipolytica* strains. Transcript levels were determined by RTqPCR, from at least biological duplicates in two technical replicates

To verify if the differences in the amounts of the produced reporter proteins result from the differences in the transcription efficiency, we analyzed the expression level of genes encoding the reporter proteins in the steady-state-maintained cells (Fig. 4). As observed, the expression level of SoA-encoding gene was the highest, irrespective of the host strain (> 4.0 log_10_RQ). No significant effect of the expression system (4UAS-pTEF in Po1h derivatives or hp4d for Po1g-derived strains) could be observed (*p* < 0.05), although each time expression of a respective gene was at least slightly higher in the Po1h host. Significantly higher expression from 4UAS-pTEF was observed for scTlG and scYFP, while comparable expression level from both promoters was observed for SoA and inYFP, which corresponds with the data on the absolute amounts of the proteins based on their reporting activity (Fig. 2e–h) and the calculated amounts of the proteins (Table 2).

## Discussion

In this study, we used chemostat cultivations as a tool to characterize heterologous protein production in *Y. lipolytica* expressing four protein-encoding genes. Two of the most widely studied and exploited *Y. lipolytica* expression platforms were used (Po1h- and Po1g-based), differing in the auxotrophy selection marker, integration site of the recombinant cassette, the promoter-governing expression of the cloned genes, and the signal peptide governing the proteins secretion (spYALI0B03564g and spXPR2pre, respectively). While indeed, the type of used auxotrophy selection marker (i.e., Leu2) was shown to impact lipids accumulation level in *Y. lipolytica* (Blazeck et al. 2014; Kerkhoven et al. 2017), no such findings were reported in terms of protein overproduction, which was the key response studied here. On the other hand, the key uncertainty of Po1h and Po1g comparison relies on the mode of the cassette integration in the genome. While the former expression system is dedicated for single-copy integrations (pBR-platform; Fig. 1) the latter one relies on random integrations using zeta elements in zeta-less strain (Pignede et al. 2000). In both cases, it was expected to obtain low-copy integrants, as non-defective selection markers were used (Le Dall et al. 1994). To account for any differences resulting from the number of the cassettes integrated with the host genome or the site of integration, several sub-clones of each type were pre-screened for growth and production of the reporters. Such strategy was previously shown to assure identification of low-copy integrants, bearing the cassette integrated at a neutral site in the genome of *Y. lipolytica* (Vogl et al. 2018; Holkenbrink et al. 2018; Theron et al. 2020). The sub-clones selected for further studies did not differ importantly in growth rate (Fig. S1), which suggests low variability in this regard coming from selection of low-copy integrants, bearing the cassette integrated at neutral site in the genome. Nevertheless, although not very probable, integration of some additional copies of the randomly integrated GGA in the Po1h genome cannot be excluded.

The stability of developed steady state in the chemostat cultures was monitored through online and off-line measurements, including DO, pH, concentration of DCW, GLY, and the major metabolites. As discussed in Arvas et al. (2011) and Liu et al. (2013), under carbon limitation, the cell growth rate, modulated by *D* [h^−1^] parameter, strongly impacts the overall physiology of a cell. In the present study, only at the highest adopted D 0.20 [h^−1^], the system exhibited satisfactory chemo-stability; although, our cultures were conducted without carbon limitation, which was the case in the referenced studies. Such *D* seems to be very high, when compared with the other reports on continuous cultures of *Y. lipolytica*—0.01 [h^−1^] (Rakicka et al. 2017), 0.009–0.031 [h^−1^] (Rywińska et al. 2011), and 0.08 [h^−1^] (Ochoa-Estopier and Guillouet 2014). However, it was evidenced that expression of the genes related to the protein synthesis is positively correlated with growth rate in *S. cerevisiae* (Castrillo et al. 2007; Liu et al. 2013) and *K. phaffii* (Buchetics et al. 2011). On the other hand, high growth rate was detrimental for production of laccase in *Y. lipolytica* (Madzak et al. 2005). In contrast, it was shown that the protein secretion process is coupled to specific growth rate in yeast, being higher at a higher growth rate. Molecular events underlying this phenomenon are endoplasmic reticulum processing, protein turnover, cell cycle, and global stress response (Liu et al. 2013). Similarly, transcriptional activity was shown to be positively correlated with increasing dilution rate in *S. cerevisiae* under carbon-limitation (Liu et al. 2013), but this observation was gene-dependent. Moreover, in *S. cerevisiae*, at lower *D* (0.05–0.10 [h^−1^]) stress-related phenomena were shown to play an important role, disabling accurate insight into the processes related to production of the heterologous proteins, which, on the other hand, played the main role at *D* > 0.10–0.20 [h^−1^] (Liu et al. 2013).

Typically, cultivations under steady state enable accurate tracking of C and N distribution between different products. While here-conducted tracking and recovery of C and N contained in the products was greatly simplified (compared with what can be achieved using C13 labelled substrate), it allowed to make some observations in this regard. Po1h_scTlG strain channeled a relatively high fraction of C to polyols at a loss in DCW, and, unlike the other strains, it produced more MAN than ERY. This strain, and the other Po1h-derivative-producing inYFP, channeled relatively high fraction of C and N to the heterologous proteins. In general, steady state of Po1g derivatives was accompanied with high accumulation of the biomass (DCW), in comparison with Po1h-derived counterparts. On the other hand, in the majority of cases, Po1h derivatives utilized higher amounts of GLY per biomass unit and uniformly produced higher amounts of the target proteins, based on their reporting activity per volume [(AAU/GAU/RFU) L^−1^]). Such an outcome could suggest that higher C flux was channeled to synthesis of the target proteins rather than biomass in Po1h derivatives. Hence, it was tempting to state that the increased specific substrate consumption and higher amount of the produced active reporter results from higher transcription efficiency of 4UAS-pTEF (Po1h) over hp4d (Po1g); although, a potential effect of the other factors differing between the two expression platforms cannot be excluded. Considering direct comparison of C and N fractions recovered in the different reporter proteins studied here, the highest amount of the elements was found in inYFP, followed by scYFP, TlG, and SoA at the end. While these differences most probably result from the mode of the proteins’ absolute amount estimation, these results well correspond with biochemical characteristics of the protein, given in Table S2. The low absolute amounts of SoA may result from high number of Cys (11), and the resulting high number of possible S–S combinations (35696; Table S2), which is the highest among the studied protein reporters. It is thus plausible, that synthesis of SoA protein is limited at disulfide bond formation in the ER, which is a stochastic process, highly demanding in terms of energy and building blocks. Limitation at this stage, could also account for the least clear difference in the extracellular SoA activity, seen between the two hosts, as ER-resident S-S forming process was the same for the two platforms, and its contribution could be higher than by the other factors. For the same reasons, i.e., only 2 Cys residues and a single predicted glycosylation site, the amounts of scYFP and inYFP reporter were the highest in terms of recovered C and N. TlG reporter also bears relatively high number of Cys residues (8; 764 possible combinations), but it is additionally highly glycosylated, which could potentially further limit its production (predicted glycosylation sites: 3 N-glycosylation, 18 O-glycosylation). On the other hand, glycosylation is a targeted process, relying on specific motives within the polypeptide structure, and thus could be less energy-consuming. Based on current results, it can be speculated that disulfide bonds formation is the most limiting step in *Y. lipolytica* host, as it strongly decreased the absolute amount of the SoA reporter, being less glycosylated and having lower molecular weight than TlG (52 vs. 65 kDa).

To get an insight into the background behind the observed lower biomass accumulation and higher production of the reporter proteins per volume in Po1h-derivative strains, expression level of the reporter-encoding genes was analyzed. Both promoters used in this study are preceded by 4 upstream activating sequences (UAS1B) (Madzak et al. 1999) which were repeatedly shown to strongly enhance transcription rate of the following gene (Blazeck et al. 2011; Blazeck et al. 2013; Dulermo et al. 2017). Upon direct comparison (Dulermo et al. 2017), 4UAS-pTEF was more transcriptionally active than hp4d, as also the core pTEF promoter is more transcriptionally active than the minimal Leu2 promoter, which is the core of hp4d (Blazeck et al. 2012). In the present study, the expression of all the heterologous genes was at least slightly higher from 4UAS-pTEF than hp4d, which corresponds to the previous literature reports and our findings on the heterologous protein production.

Linear relationships between mRNA level and the protein product are rarely seen for the secretory reporters. Such relationship was found for a relatively small CelB protein (55 [kDa]) produced in *Y. lipolytica* but was not valid when a secretory protein M1 with a higher molecular weight (120 [kDa]) was expressed in the same expression system (Swietalski et al. 2020). In the latter case, the mRNA level was disproportionally high when compared with the protein amount indicating insufficient capacity at the translation level and possible bottlenecks in folding and/or secretion. On the other hand, it was observed that highly transcribed and translated CelB could not be efficiently secreted due to overloading of the secretory pathway, as high proportion of the CelB activity was detected inside the host cells. Positive relationship between expression level and protein production and activity was identified for a panel of genes encoding both intracellular and secreted polypeptides in *Y. lipolytica*, i.e., RedStar2, glucoamylase, YFP, and α-amylase, but such linearity was unexpectedly not observed for the other secretory reporter XlnC (Dulermo et al. 2017). Consistently, production of an insulin precursor (IP) and of an amylase in *S. cerevisiae* was lower under the TEF1 promoter than under the TPI promoter even though their transcription was greater from the former (Liu et al. 2013). In all these cases, it was concluded that excessive protein production negatively affects protein folding because of the titration of chaperons and the saturation of the secretory pathway. Considering the present study, such a type of disproportion between the transcript levels and the estimated amount of the protein product was observed for the SoA reporter. Irrespective of the host strain (and the promoter used), SoA transcripts were the most abundant based on their relative quantitation. Correspondingly, no differences were observed in the amount of extracellular SoA activity between the hosts, indicating that the data corroborate themselves mutually. No intracellular activity was detected in randomly selected samples, but it is probable that SoA is active only if secreted, and cannot be detected when retained inside the cells, as found previously (Celińska et al. 2016). However, when “translated into” the absolute measures of the protein (expressed in grams and moles), these amounts were unexpectedly low. This may result from erroneous definition of a specific activity value used in the calculation, taken from a paper on native insect enzyme (Baker and Woo 1985), and/or differences in the specific activity of the native and recombinant protein. On the other hand, similar outcome was observed upon overproduction of HSA protein in *K. phaffii*, where sub-clones secreting the protein at low rate expressed the genes at significantly higher levels than the high-secreting sub-clones (Aw et al. 2017). In that study, it was evidenced that the cells overproducing secretory proteins primarily suffer from starvation, due to increased demand for nutrients upon excessive production of the heterologous protein. However, these studies were conducted in batch cultivations, where limited provision of energy and building blocks for the transcriptional and translational machinery could account for the observed lack of linearity in mRNA:protein relationship. Considering the here-adopted mode of the strains maintenance (high nutrients provision in steady state), it seems unlikely that starvation was the reason for limited production of SoA upon its efficient transcription. Aw et al. (2017) also demonstrated that high secretors exhibited decreased viability, which was also observed for the best producer and secretor (Po1h_scYFP) in this study. Furthermore, based on Po1h_scYFP example (high expression level, log_10_RQ of 3.49 vs. 4.4 for SoA, and high estimated protein production, 271.3 [μg L^−1^] vs. approx. 31 [μg L^−1^] for SoA), it seems that still sufficient capacity in translational-secretory machinery and abundance of nutrients remained available for *Y. lipolytica* host cells.

Previously, high linearity between mRNA quantity and a mean fluorescence was reported for intracellularly localized small proteins (GFP and RedStar2) expressed under a wide range of promoters of highly different strength (Blazeck et al. 2011; Dulermo et al. 2017). In this study, the levels of transcription and intracellular fluorescence were highly corresponding in both inYFP-producing strains (log_10_RQ of approx. 2.6; total [kRFU] in Po1g_inYFP, 85.278 vs. 131.509 [kRFU L^−1^] in Po1h_inYFP; [RFU] for the biomass and the supernatant is given as total). Still, as in the case of SoA, it was surprising that the expression from the weaker promoter (hp4d) is comparable with the expression governed by the stronger promoter (4UAS-pTEF). On the other hand, the expression of scYFP differed significantly, depending on the promoter type and the host strain. Higher expression of scYFP from 4UAS-pTEF was accompanied by a very high production of the protein (total in [kRFU] in the supernatant and the biomass, 722.386 vs. 20.615 for Po1h_ and Po1g_scYFP, respectively). The lower level of scYFP expression and the protein production by Po1g derivative was accompanied by expected ratio between the scYFP:inYFP (7.2), indicating efficient secretion of the reporter equipped with spXPR2, and only minor retention inside. On the other hand, the extremely high production of YFP in Po1h_scYFP was accompanied by very high retention of the YFP intended for secretion (674.000 [kRFU] in the biomass vs. 49.386 [kRFU] in the supernatant; the scYFP:inYFP ratio of 0.07). Surprisingly, intracellular accumulation of YFP in Po1h_scYFP was 5-fold higher than in Po1h_inYFP designed to achieve high intracellular [RFU] (no extracellular YFP detected). Nevertheless, it seems somehow reasonable that the strains with high secretion of a recombinant protein have high intracellular flux of this protein and thus high inFL signals than low producers. Similar observations were reported for CelB cloned in *Y. lipolytica*, which was also intended for secretion but a high proportion of the activity was detected inside the host cells (Swietalski et al. 2020). It was concluded that this high retention of CelB resulted from overloading of the secretory pathway. Corresponding conclusion could explain here observed high intracellular [RFU] of Po1h_scYFP. In our recent studies, we also observed relatively high intracellular accumulation of the fluorescent reporter targeted for secretion upon high overproduction (Gorczyca et al. 2020). On the other hand, (Theron et al. 2020) observed 7-fold lower intracellular fluorescence in the *Y. lipolytica* strains expressing GFP equipped with a signal peptide, as compared with that of strain expression intracellular variant of the fluorophore.

Still it remains puzzling what is the reason for the differences in the expression level of the genes cloned under the same promoter, or lack of significant differences in the transcripts RQ expressed from different promoters (as for SoA and inYFP). It was also observed previously that even if different genes were embedded in exactly the same expression cassettes, platforms and genetic environment, their expression level differed significantly. Such an observation was done for genes cloned in *Y. lipolytica* encoding CelB and M1 galactosidases (Swietalski et al. 2020) (3-fold higher expression level for M1 gene, at significantly lower extracellular activity), or for IP and amylase cloned in *S. cerevisiae*, where significantly higher relative transcript levels were observed for the former although both were cloned according to the same strategy (Liu et al. 2013). Nevertheless, no explanation has been postulated. It can be only speculated that either mRNA stability of the two transcripts is highly different or that transcription is somehow reversibly regulated by the translation efficiency and/or the traffic in the secretory pathway. Considering our current results, depending on the target protein characteristics, the metabolic burden imposed on the producer cell will differ (gene-to-gene variation), but also, different promoters would be differently susceptible to such feedback regulation (promoter-to-promoter variation). Definitely, more in-depth studies are required to answer these questions.

As previously postulated, depending on the characteristics of the overproduced heterologous protein, the response from the host cell in terms of its physiology and the polypeptide production rate is different. In the present study, we used steady-state maintained *Y. lipolytica* cells to investigate the impact of different heterologous proteins on the physiological behavior of the host cells. Such approach allowed to uncouple the impact of a particular protein overproduction from phenomena resulting from growth phase or caused by heterogeneity of the analyzed population. The here obtained data suggest that, using the more transcriptionally active promoter results in channeling more C flux towards the target protein, giving significantly higher specific amounts and production rates of the target polypeptide, at a loss of the biomass accumulation, with no significant impact on the polyols production. Depending on the target protein traits and the promoter governing its expression, specific responses in terms of the protein formation kinetics and the gene transcription level were observed. The same was observed previously for *S. cerevisiae* or *K. phaffii*, for which no generalizable conclusi\ons could be withdrawn, indicating strong impact of the individual combination of the target protein, promoter and some other, unidentified factors, on the host strain response. In the present study, some relationships between complexity of the reporter protein’s post-translation modifications and the absolute amounts of produced protein could be seen. Based on our current results, it can be suggested that the disulfide bonds formation in the ER is the most limiting step in *Y. lipolytica* protein production platform.

## Electronic supplementary material

ESM 1 (PDF 438 kb)
