# Supplementary material for: Impact of overproduced heterologous protein characteristics on physiological response in Yarrowia lipolytica steady-state-maintained continuous cultures
Source: Appl Microbiol Biotechnol. 2020 Oct 6;104(22):9785–800. doi: 10.1007/s00253-020-10937-w (PMC7595971; doi:10.1007/s00253-020-10937-w)
Supplement: Supplementary file 1 — (PDF 438 kb) [file 253_2020_10937_MOESM1_ESM.pdf]

Applied Microbiology and Biotechnology

Impact of overproduced heterologous proteins characteristics on physiological response in *Yarrowia lipolytica* steady-state maintained continuous cultures

Korpys-Woźniak Paulina, Kubiak Piotr, Białas Wojciech, Celińska Ewelina*

[1]Department of Biotechnology and Food Microbiology, Poznan University of Life Sciences, ul. Wojska Polskiego 48, 60-627 Poznań, Poland

*Corresponding author: Ewelina Celińska, e-mail: ewelina.celinska@up.poznan.pl, phone: +48618466007, ORCID 0000-0001-8372-8459

Fig.S1 Growth of Po1g-derived and Po1h-derived *Yarrowia lipolytica* sub-clones in shake-flask cultures of. X axis: culturing time [h], Y axis: biomass accumulation (OD600nm).

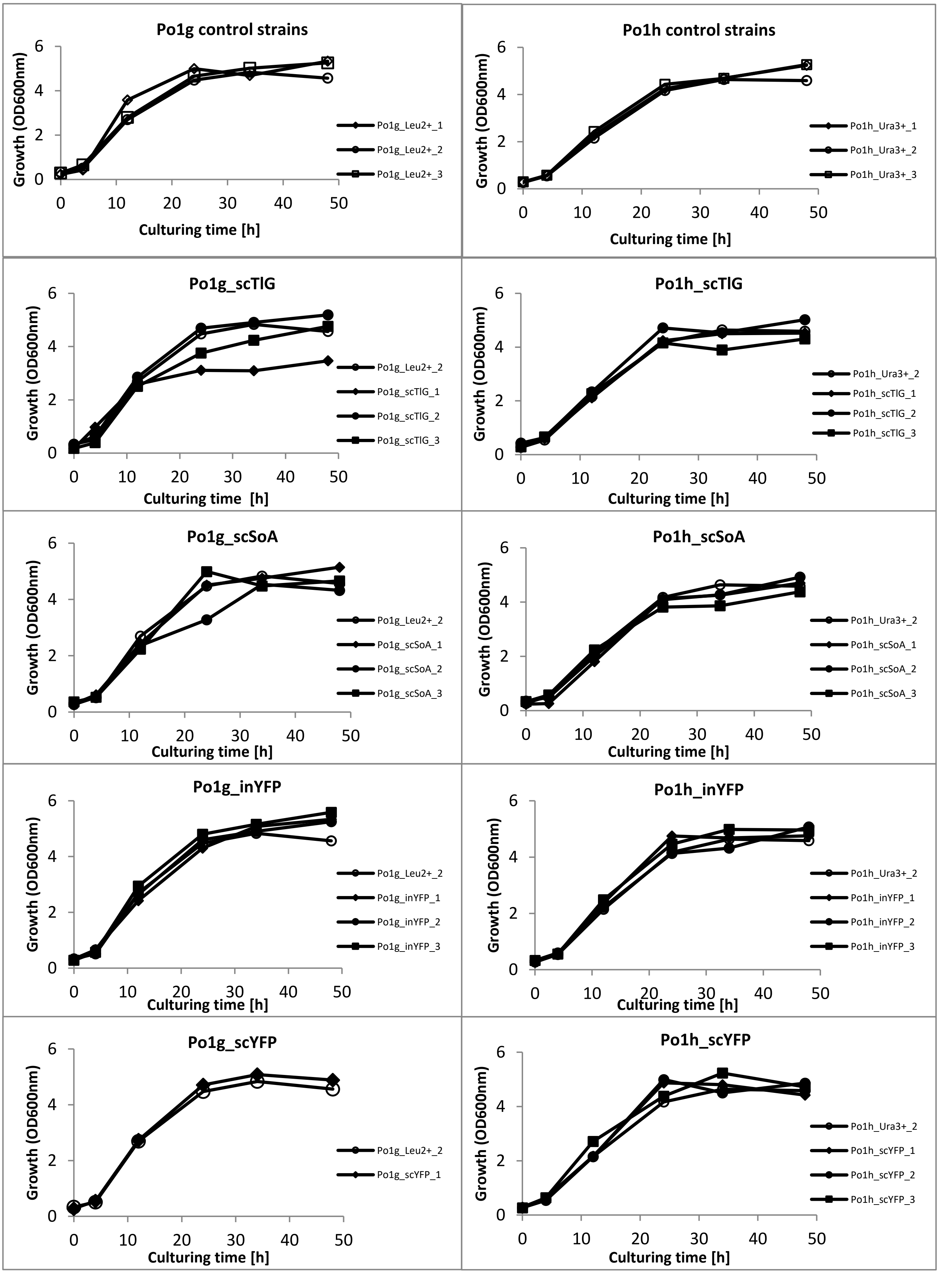

Fig.S2. Concentration of glycerol (GLY), biomass (DCW) and small molecular weight metabolites: erythritol (ERY), mannitol (MAN) and citric acid (CA) for continuous culture of *Yarrowia lipolytica* control strain (Po1h_Ura3+) grown in continuous cultures at: (A) 0.06, (B) 0.12 and (C) 0.20 [h^{-1}] dilution rates. X axis: residence time [nV], Y axis: concentration of GLY in [$g\ L^{-1}$] and DCW in [$g_{DCW}\ L^{-1}$] and ERY, MAN, CA in [$g\ L^{-1}$]. The error bars indicate ±*SD* at least duplicate.

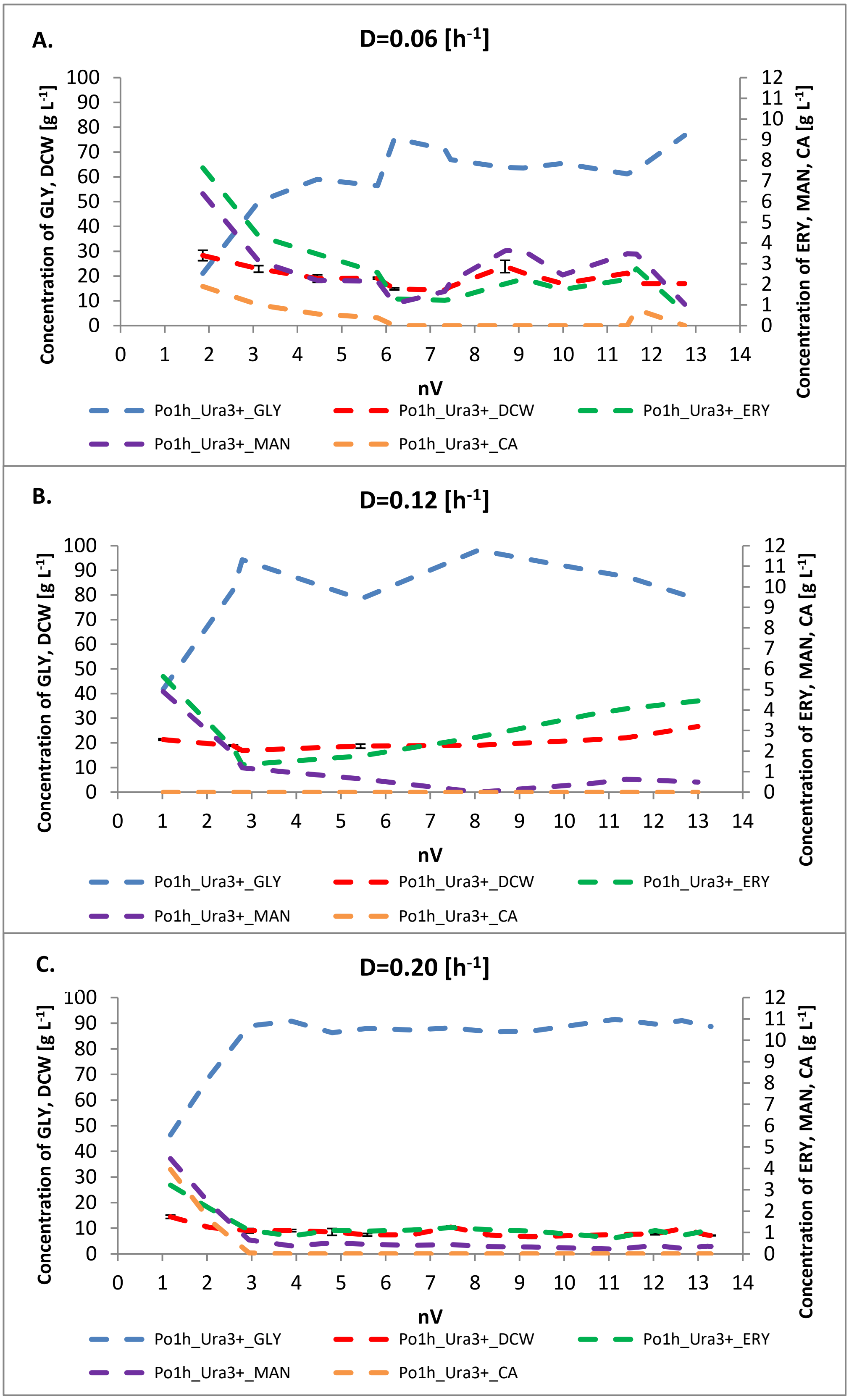

Fig.S3. Kinetics of Po1g-derived and Po1h-derived *Y. lipolytica* strains cultures, including the batch and continuous stages; X axis: culturing time (h); Y axis: glycerol concentration ([g L^{-1}]; GLY); biomass growth ([g_{DCW} L^{-1}]; DCW); erythritol [g_{DCW} L^{-1}]; ERY), mannitol ([g L^{-1}]; MAN), citric acid ([g L^{-1}]; CA), α-ketoglutaric acid ([g L^{-1}]; a-KG) concentrations in (A) Po1g_Leu2+, (B) Po1h_Ura3+,(C) Po1g_inYFP, (D) Po1h_inYFP, (E) Po1g_scYFP, (F) Po1h_scYFP, (G) Po1g_scSoA, (H) Po1h_scSoA, (I) Po1g_scTlG, (J) Po1h_scTlG strains during batch and continuous bioreactor cultures; pO_2 20%, pH 5.5; D 0.20 [h^{-1}]; Error bars indicate ±*SD* from at least biological duplicates.

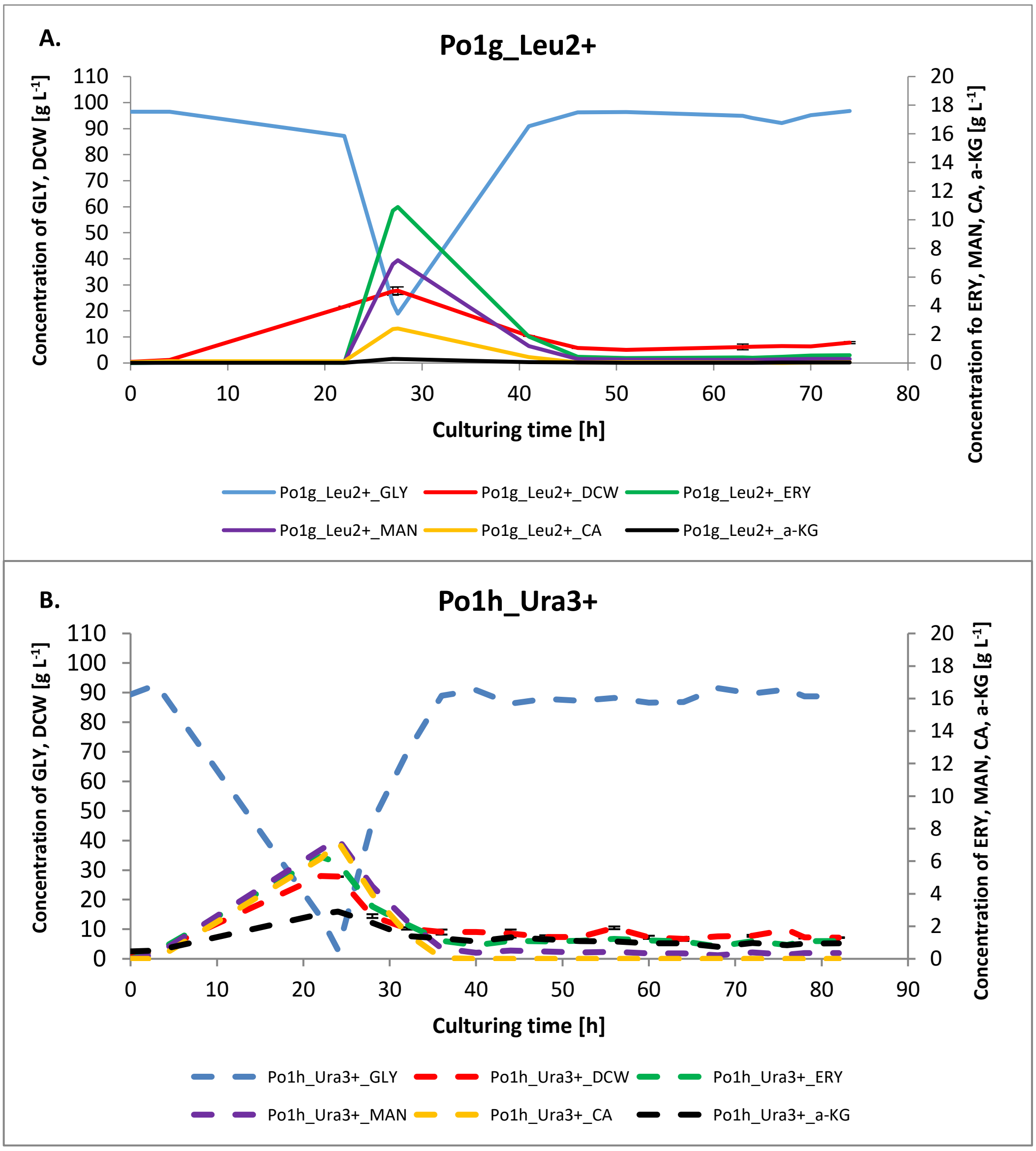

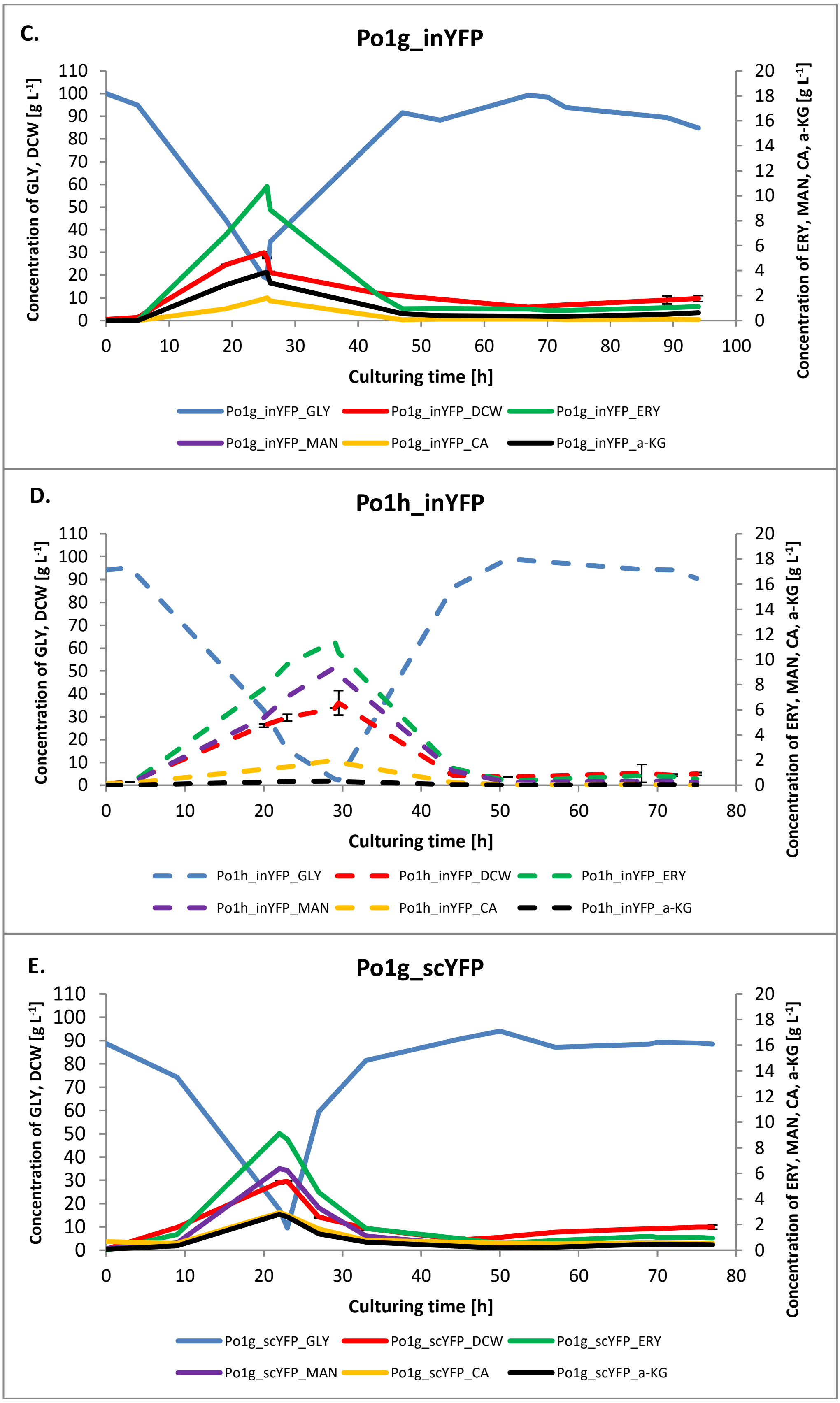

C.
Po1g_inYFP
Concentration of GLY, DCW [g L-1]
Concentration of ERY, MAN, CA, a-KG [g L-1]
110
100
90
80
70
60
50
40
30
20
10
0
20
18
16
14
12
10
8
6
4
2
0
0 10 20 30 40 50 60 70 80 90 100
Culturing time [h]
Po1g_inYFP_GLY
Po1g_inYFP_DCW
Po1g_inYFP_ERY
Po1g_inYFP_MAN
Po1g_inYFP_CA
Po1g_inYFP_a-KG
D.
Po1h_inYFP
Concentration of GLY, DCW [g L-1]
Concentration of ERY, MAN, CA, a-KG [g L-1]
0 10 20 30 40 50 60 70 80
Culturing time [h]
Po1h_inYFP_GLY
Po1h_inYFP_DCW
Po1h_inYFP_ERY
Po1h_inYFP_MAN
Po1h_inYFP_CA
Po1h_inYFP_a-KG
E.
Po1g_scYFP
Concentration of GLY, DCW [g L-1]
Concentration of ERY, MAN, CA, a-KG [g L-1]
0 10 20 30 40 50 60 70 80
Culturing time [h]
Po1g_scYFP_GLY
Po1g_scYFP_DCW
Po1g_scYFP_ERY
Po1g_scYFP_MAN
Po1g_scYFP_CA
Po1g_scYFP_a-KG

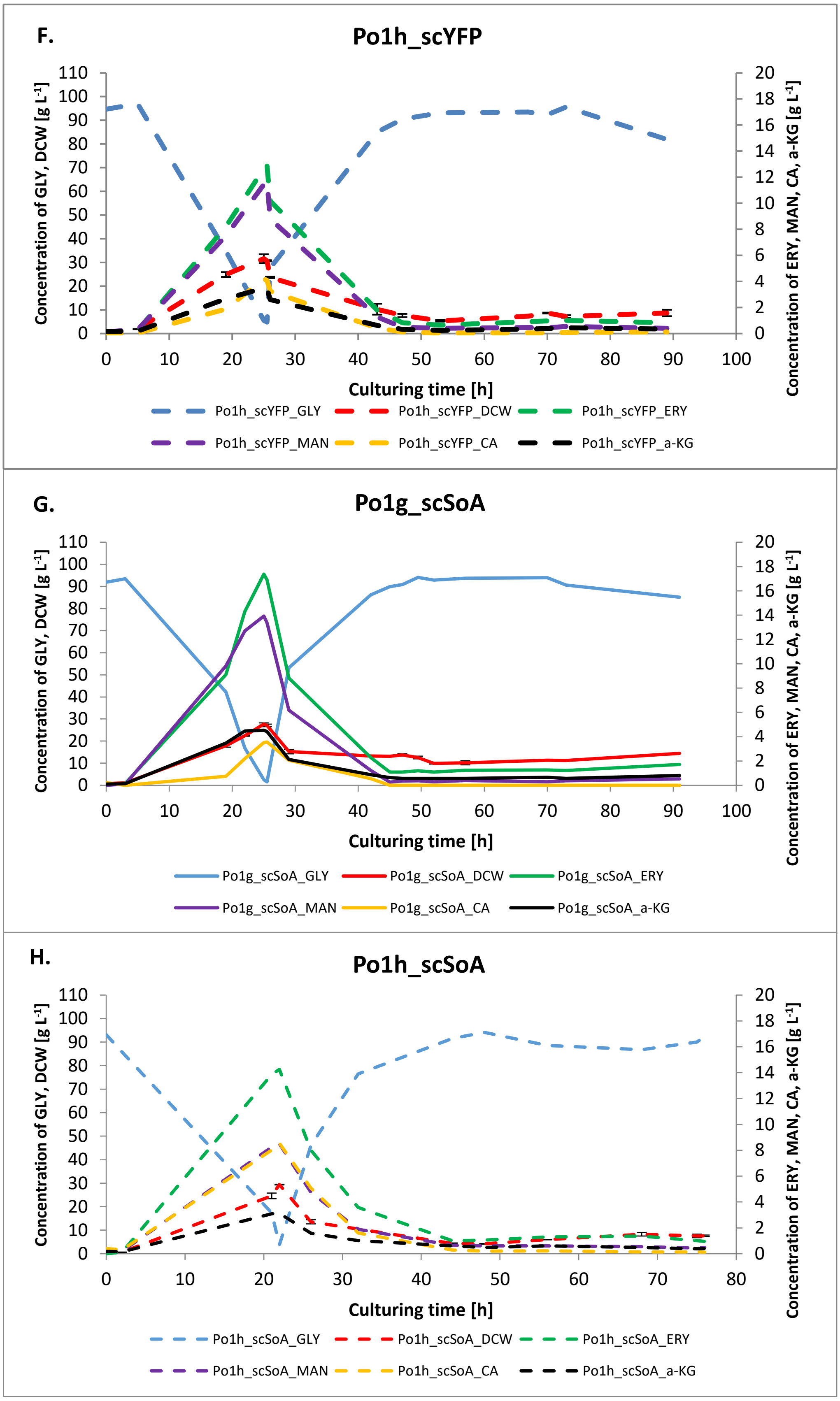
F.
Po1h_scYFP
Concentration of GLY, DCW [g L-1]
Concentration of ERY, MAN, CA, a-KG [g L-1]
Culturing time [h]
Po1h_scYFP_GLY
Po1h_scYFP_DCW
Po1h_scYFP_ERY
Po1h_scYFP_MAN
Po1h_scYFP_CA
Po1h_scYFP_a-KG
G.
Po1g_scSoA
Concentration of GLY, DCW [g L-1]
Concentration of ERY, MAN, CA, a-KG [g L-1]
Culturing time [h]
Po1g_scSoA_GLY
Po1g_scSoA_DCW
Po1g_scSoA_ERY
Po1g_scSoA_MAN
Po1g_scSoA_CA
Po1g_scSoA_a-KG
H.
Po1h_scSoA
Concentration of GLY, DCW [g L-1]
Concentration of ERY, MAN, CA, a-KG [g L-1]
Culturing time [h]
Po1h_scSoA_GLY
Po1h_scSoA_DCW
Po1h_scSoA_ERY
Po1h_scSoA_MAN
Po1h_scSoA_CA
Po1h_scSoA_a-KG

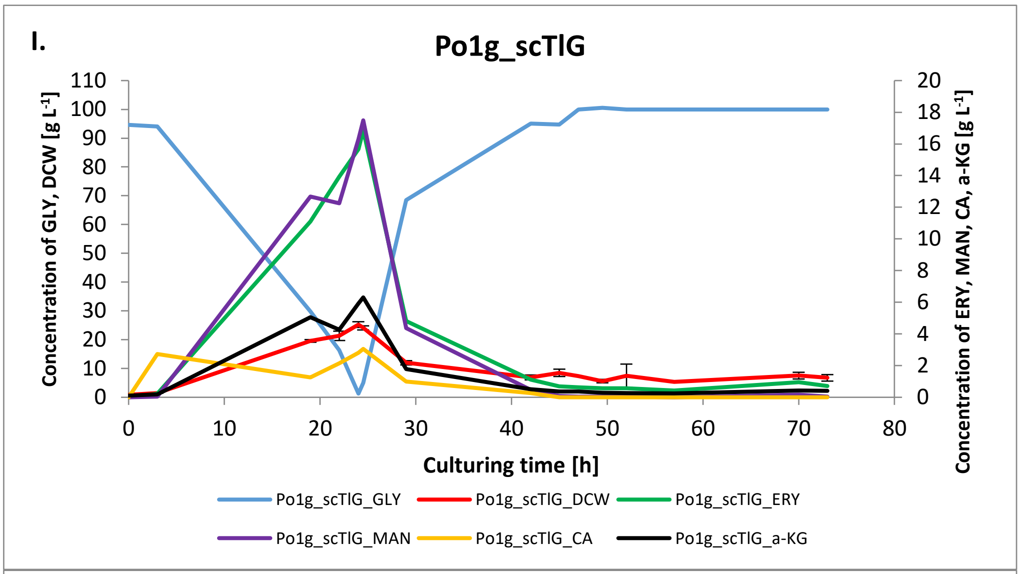
I.
Po1g_scTlG
Concentration of GLY, DCW [g L-1]
Concentration of ERY, MAN, CA, a-KG [g L-1]
Culturing time [h]
Po1g_scTlG_GLY
Po1g_scTlG_DCW
Po1g_scTlG_ERY
Po1g_scTlG_MAN
Po1g_scTlG_CA
Po1g_scTlG_a-KG

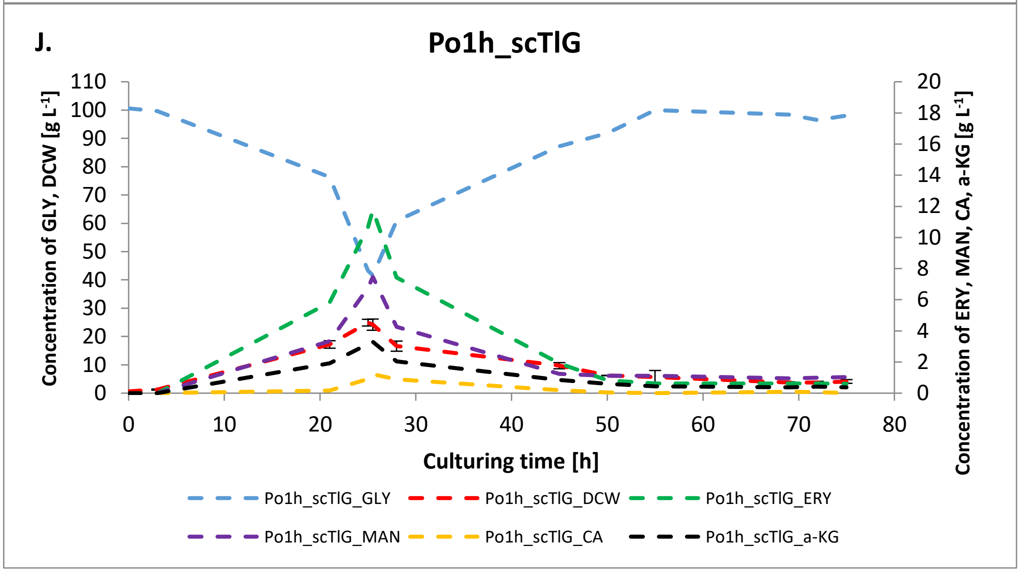
J.
Po1h_scTlG
Concentration of GLY, DCW [g L-1]
Concentration of ERY, MAN, CA, a-KG [g L-1]
Culturing time [h]
Po1h_scTlG_GLY
Po1h_scTlG_DCW
Po1h_scTlG_ERY
Po1h_scTlG_MAN
Po1h_scTlG_CA
Po1h_scTlG_a-KG

Table S1. Strains and vectors used in this study

Promoter	Name	Characteristics	Use	Supplier/reference
	**Strains**			
	***Yarrowia lipolytica* Po1g**	Genotype: *MatA, leu2-270, ura3-302::URA3, xpr2-332, axp-2*<br>Phenotype: *Leu⁻, ΔAEP, ΔAXP, Suc+, pBR platform*	Parental strain - host for expression of recombinant SoA,TlG,YFP gene	Yeastern Biotech Co.
hp4d 4UASpLeu2	*Yarrowia lipolytica* Po1g_Leu2+_2 (ECY32)	Po1g derivative transformed with Leu2 cassette	Control for Po1g derivatives	This study
	Yarrowia lipolytica Po1g_scSoA _2 (1.1.2.2)	Po1g derivative with expression cassette pYLSC_SoA	Expression of heterologous α-amylase from *S. oryzae*	Celińska et al. 2015
	Yarrowia lipolytica Po1g_scTlG _2 (2.2.1.2)	Po1g derivative with expression cassette pYLSC_TlG	Expression of heterologous glucoamylase from *T.lanuginosus*	This study
	Yarrowia lipolytica Po1g_scYFP_ 1 (ECY205)	Po1g derivative with expression cassette pYLSC_YFP	Expression of secreted heterologous YFP	This study
	Yarrowia lipolytica Po1g_inYFP_2 (ECY18)	Po1g derivative with expression cassette pYLEX_YFP	Expression of cytosolic heterologous YFP	This study
	***Yarrowia lipolytica* Po1h**	Genotype: *MatA, ura3-302, xpr2-322, axp1-2, leu2-270::LEU2*<br>Phenotype: *Ura⁻, ΔAEP, ΔAXP, Suc+*	Parental strain - host for expression of heterologous SoA,TlG,YFP gene	Celińska et al. 2020
4UASpTEF	*Yarrowia lipolytica* Po1h_Ura3+_2 (GGY231)	Po1h derivative transformed with Ura3 cassette	Control for Po1h derivatives	This study
	Yarrowia lipolytica Po1h_scSoA_1 (GGY237)	Po1h derivative with expression cassette bearing single TU with SP1 upstream fused to gene encoding α-amylase	Expression of secreted heterologous α-amylase from *S. oryzae*	This study
	Yarrowia lipolytica Po1h_scTlG_2 (GGY228)	Po1h derivative with expression cassette bearing single TU with SP1 upstream fused to gene encoding glucoamylase	Expression of secreted heterologous glucoamylase from *T.lanuginosus*	This study
	Yarrowia lipolytica Po1h_scYFP_3 (GGY251)	Po1h derivative with expression cassette bearing single TU with SP1 upstream fused to gene encoding YFP	Expression of secreted heterologous YFP	This study

	Yarrowia lipolytica<br>Po1h_inYFP_3 (GGY245)	Po1h derivative with expression cassette bearing single TU with gene encoding YFP, without SP1	Expression of cytosolic heterologous YFP	This study
	**JM109 *Escherichia coli***	F'(traD36,proAB+,lacIq,Δ(lacZ)M15), endA1,recA1,hsdR17 (rk-,mk+), mcrA,supE44,λ-gyrA96, relA1, Δ(lacproAB),thi-1	Host for routine cloning, vector assembly and propagation	Sigma-Aldrich
	**Vectors**			
hp4d 4UASpLeu2	pYLSC	pBR322 backbone, hybrid promoter (hp4d) XPR2 pre-region, leucine gene (LEU2) – selection marker Ltd, Taiwan<br>Integration site at pBR-docking platform<br>Prior to transformation the vector was linearized with NotI endonuclease http://www.yeastern.com/Products.php?pid=273	Secretion vector used for transformation of *Y. lipolytica* Po1g strain	Yeastern Biotech Co.
hp4d 4UASpLeu2	pYLEX	pBR322 backbone, hybrid promoter (hp4d), leucine gene (LEU2) – selection marker Ltd, Taiwan<br>Integration site at pBR-docking platform<br>Prior to transformation the vector was linearized with NotI endonuclease http://www.yeastern.com/Products.php?pid=273	Expression vector used for transformation of *Y. lipolytica* Po1g strain	Yeastern Biotech Co.

Table S2. Biochemical properties of the reporter proteins used in this study

Protein	Molecular weight [Da]#	Number of aa#	Theoretical pI#	Predicted N-glycosylation sites*	Predicted O-glycosylation sites**	GRAVY#	Number of Cys$	Total different combinations S-S$
**YFP**	26991.54	239	5.58	-	1	-0.464	2	2
**SoA**	52140,40	474	4.88	1	8	-0.395	11	35696
**TlG**	65154.96	600	4.90	3	18	-0.266	8	764

*http://www.cbs.dtu.dk/services/NetNGlyc/ N-glycosylation

**http://www.cbs.dtu.dk/services/NetOGlyc/ O-glycosylation

#https://web.expasy.org/protparam/ - MW, aa, GRAVY, pI

$https://npsa-prabi.ibcp.fr/cgi-bin/npsa_automat.pl?page=/NPSA/npsa_cysteines.html disulfide bonds

Table S3. Primers used in this study

Primer	Sequence 5'→3'	Reference
GGP_G1_SP1_eYFP_D_Fw	GGTCTCTAATGAAGTTCACATTTGCTGCCGTTACCGCCGCGCTGGCCTCGTCCG CCATTGCCCCAGTGAGCAAGGGCGAGGAGCTGTTCACC	This study
GGP_G1_eYFP_D_Fw	GGTCTCTAATGGTGAGCAAGGGCGAGGAGCTGTTCACC	
GGP_G1_eYFP_E_Rev	GGTCTCTTAGATTACTTGTACAGCTCGTCCATGCCGAG	
GGP_G1_SP1_D_Fw	GGTCTCTAATGAAGTTCACATTTGCTGCCG	Celińska et al. 2017
GGP_G1_SoA_E_Rev	GGTCTCTTAGACTAGTGGTGGTGGTGG	This study
GGP_G1_TlG_E_Rev	GGTCTCTTAGATCAATGATGATGATGATGATG	
GGP_T1_LIP2_E_Fw	GGTCTCTTCTAGTGTCTGTGGTATCTAAGCTATTTATC	
GGP_T1_LIP2_F_Rev	GGTCTCTAAGCCGATTTGTCTTAGAGGAACGC	
GGP_P1_4UASpTEF_GS_C_Fw	GGGGTCTCTAGCTGCATGCTGAGGTGTCTCACAAG	
GGP_P1_4UASpTEF_GS_D_Rev	GGTCTCTCATTCTTCGGGTGTGAGTTGACAAGG	
GGP_M_Ura3_B_Fw	GGTCTCTAGGTATAACTTCGTATAGCATACATTATACGAAG	
GGP_M_Ura3_C_Rev	GGTCTCTAGCTTCGCTTCGGATAACTCC	
GGP_InsertUP_zeta_A_Fw_*Not*I	GGTCTCTGCCTGCGGCCGCTGTCGGGAACCGC	
GGP_InsertUP_zeta_B_Rev	GGTCTCTACCTTCTAGCAAAGTGCTTTGTGC	
GGP_InsertDOWN_*zeta*_L_Fw	GGTCTCTGAGTCATGTGTAACACTCGCTCTG	
GGP_InsertDOWN_*zeta*_M_Rev_*Not*I	GGTCTCTCGCAGCGGCCGCACTGAAGGGCTTTGTGAG	
6560 F	GAT CCG GCA TGC ACT GAT C	Yeastern Biotech Co.
6904 R	AAC ACC GGT GTT GGA CTC AG	
pYLSC_YFP_*KpnI*_Rev	AAAGGTACCTTACTTGTACAGCTCGTCCATGCCG	This study
pYLSC_YFP_*SfiI*_Rev	GGCCGTTCTGGCCCATGGTGAGCAAGGGCGAGGAGCTG	
pYLEX_YFP _Fw	AATGGTGAGCAAGGGCGAGGAGCTGTTCACCG	
pYLSC/EX_YFP_*Kpn*I_Rev	AAAGGTACCTTACTTGTACAGCTCGTCCATGCCG	
pYLSC _SoA_ *Sfi*I _Fw	A*GGCCGTTCTGGCC*CAGAAGGACCCCCACTTCC	Celińska et al. 2015
pYLSC_SoA_*Kpn*I_Rev	AAGGTACC CTAGTGGTGGTGGTGGTGGTG	
pYLSC_TlG_*Sfi*I_Fw	A*GGCCGTTCTGGCC*AGACCAGAAACAACCGTTCC	This study
pYLSC_TlG_*Kpn*I_Rev	AAGGTACC TCAATGATGATGATGATGATG	
r-t_SoA_Fw	GTAACAACGTGGGAATCCGAAT	
r-t_SoA_Rev	CCCTGGCCGTTCGAAGTAG	
r-t_TlG_Fw	TCTGATGTTGTTTGGGAATCTGA	
r-t_TlG_Rev	TGATGTCTCCACCAAGAGTTTACTG	
r-t_YFP_Fw	CAACAGCCACAACGTCTATATCATG	
r-t_YFP_Rev	ATGTTGTGGCGGATCTTGAAGT	
r-t_ACT_Fw	CGAGCGAATGCACAAGGA	
r-t_ACT_Rev	GAGCGGTGATCTTGACCTTGA	

Table S4. Viability of Po1g- and Po1h-derived *Y. lipolytica* strains maintained in steady-state in chemostat cultivations. ([*]- surface colony forming unit).

Strains	[scfu*]
Po1g_Leu2+	8.30*10^4
Po1h_Ura3+	1.93*10^5
Po1g_inYFP	1.43*10^5
Po1h_inYFP	1.77*10^5
Po1g_scYFP	1.10*10^5
Po1h_scYFP	9.70*10^4
Po1g_scSoA	1.00*10^5
Po1h_scSoA	1.67*10^5
Po1g_scTlG	1.70*10^5
Po1h_scTlG	1.07*10^5
